# Parathyroidectomy versus cinacalcet for tertiary hyperparathyroidism; a retrospective analysis

**DOI:** 10.1007/s00423-019-01755-4

**Published:** 2019-02-07

**Authors:** R. R. Dulfer, E. Y. Koh, W. Y. van der Plas, A. F. Engelsman, E. J. M. Nieveen van Dijkum, R. A. Pol, L. Vogt, M. H. de Borst, S. Kruijff, A. Schepers, N. M. Appelman-Dijkstra, J. I. Rotmans, D. A. Hesselink, C. H. J. van Eijck, E. J. Hoorn, T. M. van Ginhoven, on behalf of the Dutch Hyperparathyroid Study Group

**Affiliations:** 1 Department of Surgery, Erasmus MC, University Medical Center Rotterdam, ‘s-Gravendijkwal 230, P.O. Box 2040, 3000 CA Rotterdam, The Netherlands; 2 Department of Surgery, Academic Medical Center, University of Amsterdam, Amsterdam, The Netherlands; 3 Department of Surgery, University Medical Center Groningen, University of Groningen, Groningen, The Netherlands; 4 Department of Nephrology, Academic Medical Center, University of Amsterdam, Amsterdam, The Netherlands; 5 Department of Nephrology, University Medical Center Groningen, University of Groningen, Groningen, The Netherlands; 6 Department of Surgery, Leiden University Medical Center, Leiden, The Netherlands; 7 Department of Internal Medicine, Leiden University Medical Center, Leiden, The Netherlands; 8 Department of Internal Medicine, Division of Nephrology and Kidney Transplantation, Erasmus MC, University Medical Center Rotterdam, Rotterdam, The Netherlands

**Keywords:** Cinacalcet, CKD-BMD, Parathyroidectomy, Tertiary hyperparathyroidism

## Abstract

**Introduction:**

Tertiary hyperparathyroidism (tHPT), i.e., persistent HPT after kidney transplantation, affects 17–50% of transplant recipients. Treatment of tHPT is mandatory since persistently elevated PTH concentrations after KTx increase the risk of renal allograft dysfunction and osteoporosis. The introduction of cinacalcet in 2004 seemed to offer a medical treatment alternative to parathyroidectomy (PTx). However, the optimal management of tHPT remains unclear.

**Methods:**

A retrospective analysis was performed on patients receiving a kidney transplantation (KT) in two academic centers in the Netherlands. Thirty patients undergoing PTx within 3 years of transplantation and 64 patients treated with cinacalcet 1 year after transplantation for tHPT were included. Primary outcomes were serum calcium and PTH concentrations 1 year after KT and after PTx.

**Results:**

Serum calcium normalized in both the cinacalcet and the PTx patients. PTH concentrations remained above the upper limit of normal (median 22.0 pmol/L) 1 year after KT, but returned to within the normal range in the PTx group (median 3.7 pmol/L). Side effects of cinacalcet were difficult to assess; minor complications occurred in three patients. Re-exploration due to persistent tHPT was performed in three (10%) patients.

**Conclusion:**

In patients with tHPT, cinacalcet normalizes serum calcium, but does not lead to a normalization of serum PTH concentrations. In contrast, PTx leads to a normalization of both serum calcium and PTH concentrations. These findings suggest that PTx is the treatment of choice for tHPT.

## Introduction

Hyperparathyroidism (HPT) is a state of overproduction of parathyroid hormone (PTH) due to over activity of one or more of the parathyroid glands [1]. Secondary HPT (sHPT) frequently complicates chronic kidney disease (CKD). CKD causes phosphate retention, hypovitaminosis D [1,25-(OH)_2_-D], and hypocalcemia, leading to stimulation of the parathyroid glands and increased PTH production. Secondary HPT occurs in 25–30% of patients with end-stage renal disease (ESRD) and is associated with severe bone mineral disease [2]. Importantly, sHPT is also associated with increased cardiovascular morbidity [2] and mortality [3, 4]. The first line of treatment of sHPT used to consist of calcium and vitamin D supplementation followed by either medical therapy using calcimimetics [5] or surgical treatment by parathyroidectomy (PTx) [6]. For dialysis patients requiring PTH-lowering therapy, the current recommendation is to treat with calcimimetics, calcitriol or vitamin D analogs, or a combination of these therapies [7].

Secondary HPT can be corrected by a successful kidney transplantation (KT) [8]. Nevertheless, 17–50% of patients who receive a kidney transplant remain in a state of HPT 1 year after KT [9–11]. This is referred to as tertiary or post-renal transplantation HPT (tHPT), which is characterized by excessive and autonomous production and excretion of PTH [8, 12]. In general, the PTH concentration rapidly declines within the first 3 months after KT and then continues to decline more gradually over the course of the next 9 months. One year after transplantation, PTH concentrations are unlikely to decline further [9–11].

As tHPT increases the risk of renal allograft dysfunction and renal allograft loss [13, 14], osteoporosis [15], and bone fractures [16], adequate management is essential. Introduced in 2004, the calcimimetic drug cinacalcet (Mimpara^®^_,_ Amgen Inc., Thousand Oaks, CA, USA) is frequently used for the treatment of ESRD-related HPT [17, 18]. Cinacalcet suppresses the production of PTH by increasing the sensitivity of the calcium-sensing receptor of the parathyroid gland to calcium [19]. The United States Food and Drug Administration and the European Medicines Agency have approved cinacalcet for the treatment of secondary but not tertiary HPT. This is despite a number of small non-randomized studies demonstrating the safety of cinacalcet in tHPT, with gastrointestinal intolerance being the most common side effect [20].

Before the introduction of cinacalcet, the only treatment option for patients with tHPT was parathyroidectomy (PTx) [21]. This surgical procedure involves either subtotal or total PTx (with or without auto-transplantation), which are both effective and safe procedures [22, 23]. In sHPT, PTx was even associated with a lower risk of major cardiovascular events after surgery [24].

The effect of cinacalcet on cardiovascular morbidity and mortality has recently been studied in the EVOLVE trial [25]. This study in dialysis patients demonstrated that treatment with cinacalcet, despite lowering serum calcium in a sufficient way, does not reduce the risk of major cardiovascular events or death. In an additional meta-analysis of randomized controlled trials, cinacalcet did not improve overall survival of dialysis patients [26]. Consequently, the Australian Government stopped the reimbursement of cinacalcet (https://www.nps.org.au/radar/articles/sensipar-cinacalcet-pbs-listing-to-be-deleted#article).

The only randomized controlled trial comparing the effects of PTx and cinacalcet in the treatment of tHPT included 30 patients and concluded that 66% of patients treated with cinacalcet achieved normocalcemia compared with 100% after PTx [27]. As this is the only trial comparing these treatment modalities and having a small sample size, the optimal management strategy for tHPT remains unclear and additional studies including larger patient numbers are needed. In this study, the outcomes of treatment with PTx or cinacalcet for tHPT were compared in a larger cohort of patients derived from the data of two academic centers.

## Methods

### Study population

All patients who received a first KT at one of the two participating university medical centers between 1994 and 2015 and had a history of PTx or calcimimetic use, both before and after KT, were included. Their demographic and clinical data were stored in a central database. This study was approved by the institutional review boards of all participating centers.

Inclusion criteria for this study were a diagnosis of tHPT, age ≥ 18 years at the time of KT, and the availability of serum calcium and serum PTH concentrations after KT. Tertiary HPT was defined as the need for PTx within 3 years after KT or the (continuous) need of cinacalcet treatment at month 12 after KT. Patients with previous parathyroid surgery were excluded. Two groups were identified. Group 1 (cinacalcet group) consisted of patients who received treatment with cinacalcet at month 12 after their first KT without a history of PTx. Group 2 (PTx group) consisted of patients who underwent a PTx within 3 years of their first KT. Outcomes of the PTx group in the first year after KT are presented for patients who had not undergone PTx at the set time points.

The primary outcomes were serum calcium and PTH concentration 1 year after KT and 1 year after PTx. Secondary outcomes were the use of co-medication (calcium supplementation, vitamin D supplementation, cinacalcet) after KT and PTx and complications after KT and PTx.

### Data collection

Electronic patient files were reviewed for patient data. Baseline characteristics were gender, age, primary kidney disease, type and duration of dialysis, medication use, and relevant co-morbidity (e.g., diabetes mellitus and cardiovascular disease).

KT data included donor type and age, ischemia times, post-operative complications graded by the Clavien-Dindo scale [28], delayed graft function (defined as the need for dialysis in first week after KT), primary non-function, biochemical parameters until 5 years after KT (calcium, phosphate, albumin, creatinine, alkaline phosphatase, and PTH), and medication use after KT (calcium or vitamin D supplementation, phosphate binders, and calcimimetics). Data on the use of diuretics were not systematically collected.

PTx data included type of the surgical procedure (total or subtotal), complication rate, imaging before PTx, laboratory data before PTx until 5 years after PTx (calcium, phosphate, albumin, creatinine, alkaline phosphatase and PTH), and medication use after PTx (calcium or vitamin D supplementation, phosphate binders, and calcimimetics). The decision to perform a total or subtotal parathyroidectomy was at the surgeons’ discretion. There was no routine use of neuromonitoring and no routine post-operative laryngoscopy.

Serum calcium concentrations were adjusted for albumin according to the following formula: adjusted total calcium (mmol/L) = measured calcium (mmol/l) + (0.025 × (40 − [albumin (g/L)])). The reference value for calcium was 2.20–2.60 mmol/L. The reference value for PTH was 1.4–7.3 pmol/L. PTH values were measured with the Vitros ECi assay (Ortho Diagnostics) and with the Roche Cobas assay (Roche). The reference values for creatinine were 65–115 μmol/L for male patients and 55–90 μmol/L for female patients. Persistent post-operative hypocalcemia was defined as the need for calcium supplementation 6 months after PTx.

### Statistical analysis

Distribution was assessed using the Shapiro-Wilk test for normality. Continuous variables are presented as median with interquartile range (IQR), and categorical variables were described as count (*n*) and percentage (%). Differences between the two groups were analyzed using the Mann-Whitney *U* test for continuous variables and the Pearson chi-square or Fisher’s exact test for nominal variables. The Wilcoxon signed-rank test was used to compare the differences between time points. Statistical analysis was performed using IBM SPSS Statistics 21 software (IBM Corp., Chicago, IL, USA).

## Results

### Study population

**Fig. 1:**
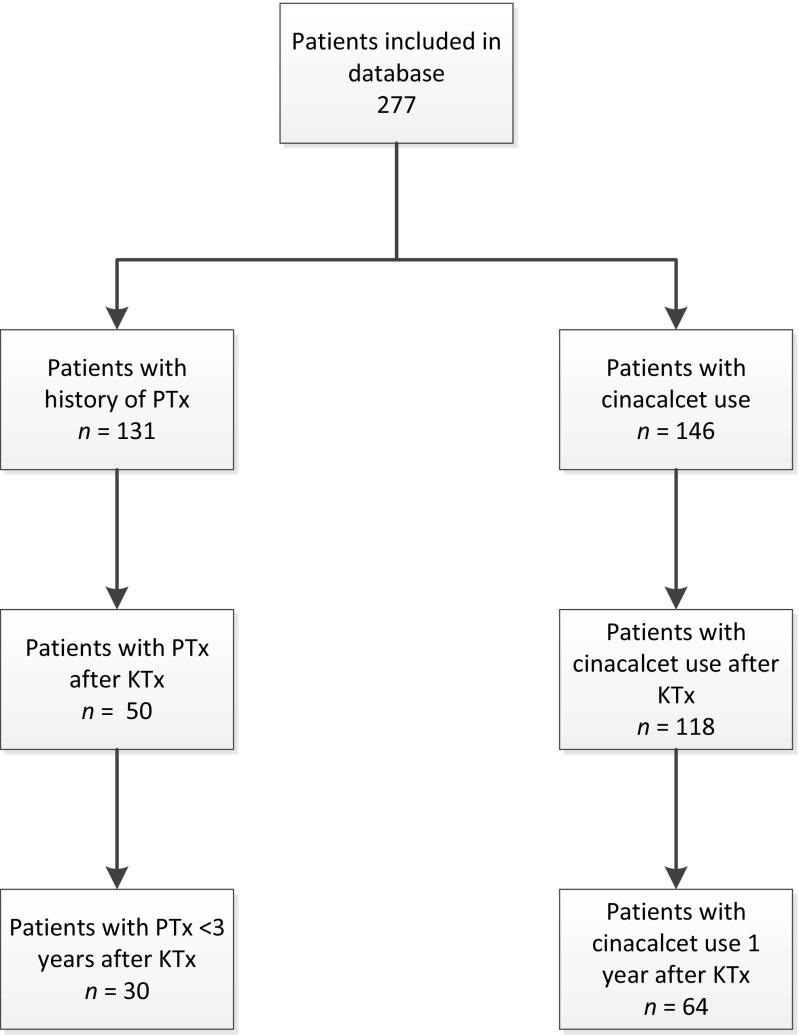
Flowchart of patient selection

A total of 277 patients were included in our database. A total of 94 patients (33.9%) were included in this study based on the abovementioned inclusion and exclusion criteria. Thirty patients were included in the PTx group whereas 64 patients were included in the cinacalcet group. A flowchart of patient selection is depicted in Fig. 1.

### Baseline characteristics

Patient characteristics at the time of KT are listed in Table 1. Median age of all patients at time of KT was 56.0 years (45.8–62.0). The median age of PTx patients at time of KT was lower (52.5 years (35.8–57.3)) compared with that of patients using calcimimetics (59.0 years (50.0–64.0), *p* = 0.034). The median duration of dialysis before KT was 42.5 months (21.0–63.0); 43.6% of patients were female and 25.5% of patients had a history of diabetes mellitus. At the time of KT, 81.3% of patients received phosphate binders, 66.0% received vitamin D supplements, 23.3% received calcium supplementation, and 45.7% received calcimimetics. Of all KT, 55.3% was performed with a deceased donor kidney. Delayed graft function occurred in 27.7% of patients. Cold and warm ischemia times were significantly longer in the PTx group (23 versus 15 h, *p* = 0.023, and 29 versus 23.5 min, *p* = 0.048, respectively).

**Table 1:** Baseline characteristics

	Cinacalcet group	PTx group	*p* value
Characteristic			
No. of patients	64	30	
Age at start dialysis (years)	53.5 (45.3–61.8)	49.5 (32.8–55.8)	0.034
Sex (female)	27 (42.2)	14 (46.7)	NS
Charlson score	3 (2–5)	3 (2–4)	NS
Dialysis type			NS
- No dialysis	12 (18.8)	2 (6.7)	
- Peritoneal dialysis	19 (29.7)	11 (36.7)	
- Hemodialysis shunt	29 (45.3)	15 (50.0)	
- Hemodialysis line	4 (6.3)	2 (6.7)	
Duration of dialysis (months)	42.5 (21.3–67.5)	42.5 (18–54.8)	NS
History of diabetes	15 (23.4)	9 (30.0)	NS
Use of medication before KT			
- Vitamin D	47 (74.6)	15 (53.6)	NS
- Phosphate binders	52 (82.5)	22 (78.6)	NS
- Calcium suppletion	12 (19)	9 (32.1)	NS
- Cinacalcet	38 (60.3)	5 (17.2)	< 0.001
Transplant characteristics			
Age at transplantation (years)	59.0 (50.0–64.0)	52.5 (35.8–57.3)	0.034
Donor type			NS
- DCD	17 (26.6)	8 (26.7)	
- DBD	18 (28.1)	9 (30.0)	
- LR	13 (20.3)	6 (20.0)	
- LUR	16 (25.0)	7 (23.3)	
Complications			NS
- Grade 2	16 (25.0)	9 (30.0)	
- Grade 3	6 (9.4)	6 (20)	
Delayed graft function	19 (29.7)	7 (23.3)	NS
Primary non-function	0 (0)	2 (6.7)	NS
Cold ischemia time (h)	15 (12–19.8)	21 (16–24)	0.023
Warm ischemia time (min)	23.5 (18–32)	29 (22–35)	0.048

Data are expressed as median (interquartile range) or *n* (%)

*PTx* parathyroidectomy

### Outcomes after kidney transplantation

Pre- and post-KT laboratory values are listed in Table 2. Serum calcium concentrations were higher in the PTx group preoperatively (2.51 versus 2.42 mmol/L). There was a significant increase in serum calcium concentrations at 3 months after transplantation in both groups (cinacalcet group: *p* = 0.002; PTx group: *p* = 0.001), but this did not persist after 6 months in the cinacalcet group (*p* = 0.148). PTx patients not yet operated in the first post-transplant year remained hypercalcemic during the first post-transplant year. Calcium concentrations in the PTx group were higher at all time points, with a median calcium of 2.76 mmol/L 1 year after transplantation.

**Table 2:** KT outcomes

	Group 1 (cinacalcet)	Group 2 (PTx)	*p* value	Number at risk PTx
*N*	64	30		
Corrected calcium (mmol/L)				
Pretransplantation	2.42 (2.23–2.54)	2.51 (2.33–2.69)	0.030	30
3 months	2.50 (2.36–2.63)	2.84 (2.64–2.90)	< 0.001	30
6 months	2.44 (2.34–2.59)	2.79 (2.65–2.85)	< 0.001	25
1 year	2.40 (2.23–2.49)	2.76 (2.56–2.90)	< 0.001	18
PTH (pmol/L)				
Pretransplantation	54.4 (29.7–91.0)	90.0 (61.4–156.4)	0.013	30
3 months	22.7 (14.0–32.7)	34.3 (25.3–80.3)	0.007	30
6 months	18.2 (14.0–27.9)	26.3 (16.3–55.1)	0.056	25
1 year	22.0 (13.7–33.1)	29.8 (15.3–57.1)	NS	18
Creatinine (μmol/L)				
3 months	119 (103–163)	126 (112–179)	NS	30
6 months	125 (100–164)	118 (100–142)	NS	25
1 year	122 (101–161)	127 (102–173)	NS	18
Use of vitamin D				
Pretransplantation	47 (74.6)	15 (53.6)	NS	30
3 months	15 (26.8)	3 (10.3)	NS	30
6 months	9 (15.8)	2 (8.3)	NS	25
1 year	9 (14.1)	2 (11.1)	NS	18
Use of cinacalcet				
Pretransplantation	38 (60.3)	5 (17.2)	< 0.001	30
3 months	38 (67.9)	2 (6.9)	< 0.001	30
6 months	50 (87.7)	3 (12.5)	< 0.001	25
1 year	64 (100)	3 (17.6)	< 0.001	18

Data are expressed as median (interquartile range) or *n* (%)

*PTx* parathyroidectomy

Median PTH concentrations were significantly higher in the PTx group before KT (90.0 versus 54.4 pmol/L, *p* = 0.013) and 3 months after transplantation (34.3 versus 22.7 pmol/L, *p* = 0.007). Both groups showed a decline in serum PTH concentrations after kidney transplantation. In the PTx group, this decline was statistically significant at 6 months after transplantation (*p* = 0.012) but not observed at 1 year post KT. In the cinacalcet group, PTH concentrations declined significantly after KT at all time points (*p* < 0.001 at all time points). In the PTx group, 10.3% of patients used cinacalcet 1 year after transplantation. In total, 34.5% of patients in the PTx group used vitamin D analogs 1 year after transplantation, whereas 14.1% of patients in the cinacalcet group used vitamin D analogs.

### Parathyroidectomy

The characteristics of the patients who underwent PTx are listed in Table 3. Median age at the time of PTx was 54.5 years; 66.7% of patients were classified as ASA III. Twelve patients (40%) underwent a PTx in the first year, and 18 underwent (60%) PTx in the second or third year after KT. Subtotal parathyroidectomy was performed in 83.3% of patients. Re-exploration due to persistent tHPT was performed in three (10%) patients. Initial operation was subtotal PTx in two patients and a less than subtotal PTx in one patient. Two patients required a second operation within the first post-operative month and one 10 months after initial surgery due to persistent HPT. There were no post-operative hematomas requiring re-exploration, no recurrent laryngeal nerve injuries, and no post-operative cardiovascular events, and there was no post-operative mortality. Post-operative hypocalcemia was apparent in 40% of patients.

**Table 3:** PTx characteristics

Characteristic	PTx group
Age at parathyroidectomy (years)	54.5 (36.8–59.3)
ASA classification	
II	10 (33.3)
III	20 (66.7)
Preoperative imaging	
No imaging	18 (62.1)
Ultrasound	4 (13.8)
MIBI scan	3 (10.3)
Ultrasound and MIBI scan	4 (13.8)
Type of PTx	
Total parathyroidectomy	3 (10.0)
Subtotal parathyroidectomy	25 (83.3)
Other	2 (6.7)
Complications	
Post-operative hypocalcemia	12 (40)
Recurrent laryngeal nerve damage	0 (0)
Surgical site infection	1 (3.3)
Pneumonia	1 (3.3)
ICU admission	1 (3.3)
Mortality	0 (0)
Re-exploration	3 (10)
Weight of parathyroid glands (g)	1.9 (1.2–3.0)

Data are expressed as median (interquartile range) or *n* (%)

*PTx* parathyroidectomy

Laboratory values before and after PTx are listed in Table 4. Serum calcium concentrations declined significantly after PTx; no patients were hypercalcemic 1 year after PTx. PTH concentrations declined significantly after PTx and were in the reference range. Vitamin D analogs were used in 42.9% of patients 1 year after PTx. One patient used cinacalcet (3.6%) after PTx. A total of 40% of patients used calcium supplementation 6 months after surgery.

**Table 4:** PTx outcomes

	PTx group	*p* value^
Corrected calcium (mmol/L)		
Preoperative	2.76 (2.62–3.04)	
3 months	2.30 (2.23–2.43)	< 0.001
6 months	2.31 (2.16–2.37)	< 0.001
1 year	2.34 (2.14–2.41)	< 0.001
PTH (pmol/L)		
Preoperative	35.5 (20.3–62.8)	
3 months	9.0 (4.7–20.8)	0.001
6 months	9.5 (3.3–22.6)	0.009
1 year	3.7 (1.2–9.3)	0.012
Creatinine (μmol/L)		
Preoperative	122 (104–173)	NS
3 months	141 (107–177)	0.015
6 months	147 (121–214)	NS
1 year	140 (107–214)	NS
Use of vitamin D		
3 months	14 (50.0)	
6 months	15 (53.6)	
1 year	12 (42.9)	
Use of cinacalcet		
3 months	1 (3.6)	
6 months	1 (3.6)	
1 year	1 (3.6)	

Data are expressed as median (interquartile range) or *n* (%)

*PTx* parathyroidectomy

^*p* value: compared with preoperative measurements

## Discussion

This study describes a cohort of patients treated for tHPT, either by PTx or by cinacalcet. After PTx, patients were normocalcemic and PTH concentrations normalized. Treatment with cinacalcet resulted in normocalcemia but not in normalization of PTH concentrations. After kidney transplantation, the PTH concentrations declined in both groups, but were higher in the group of patients that would still undergo PTx. Median PTH concentrations in both groups remained above the upper limit of normal (7.3 pmol/L) up to 12 months after transplantation. In the cinacalcet group, the serum calcium concentrations remained normal, whereas patients who would undergo PTx became hypercalcemic. After parathyroidectomy, both serum calcium concentrations and PTH concentrations were in the normal range. No differences in renal transplant function were observed, despite significantly longer ischemia times in the PTx group.

These findings raise questions about the efficacy of off-label use of cinacalcet in the treatment of tertiary hyperparathyroidism. Adequate control of PTH in the post-transplant period is paramount given the increased risk of allograft dysfunction, allograft loss 13, 14, all-cause mortality 30, fractures, and osteoporosis [15, 16]. These reservations concerning the treatment of tHPT with cinacalcet are also supported by a study of Cruzado and colleagues [27]. In this study, patients with tHPT were randomized between PTx and cinacalcet therapies. The primary outcome was achievement of normocalcemia after 1 year. In patients with a PTx, 100% achieved normocalcemia, in comparison with 67% of patients after treatment with cinacalcet.

Patients with ESRD are fragile as shown from high ASA scores, and it is therefore understandable that there is some reluctance to refer patients for surgical treatment [29]. Still, in this study, surgical complications after PTx were rare. Cardiovascular events did not occur and only one patient developed pneumonia. There were no clinical recurrent laryngeal nerve injuries, although only patients with dysphonia underwent laryngoscopy. This is comparable with the results of another large Dutch study [30]. The rate of post-operative hypocalcemia in this study was 40% at 6 months. This was higher than expected in view of results from older studies [31, 32]. However, patients in our PTx group were mainly hypercalcemic patients, who more frequently have hungry bone syndrome and require longer calcium supplementation. Post-operative increase in serum creatinine levels was seen at 3 months after PTx. At 12 months, there were no statistical differences compared with baseline value. A temporary decline in graft function is seen often after PTx; however, it does not influence graft survival [33]. Overall, PTx is a safe procedure, even in this fragile population [22, 30, 32, 34].

In our study, both total and subtotal parathyroidectomies were performed, which are both safe and effective procedures [23]. A randomized controlled trial comparing these procedures in sHPT found similar results for both techniques with a slightly higher rate of recurrence after subtotal parathyroidectomy [31]. Such a study has not been performed for tHPT [35]. However, favorable results have been reported concerning subtotal parathyroidectomy for tHPT, albeit not in an RCT [22, 27]. Considering that the underlying metabolic disorders responsible for the occurrence of HPT have been corrected after successful transplantation, we prefer subtotal parathyroidectomy. This procedure is associated with a lower risk of post-operative hypocalcemia and persistent hypoparathyroidism while recurrences are unlikely due to the metabolic changes.

This study was not designed to evaluate cost-effectiveness and therefore we cannot report on this. To date, no studies evaluating cost-effectiveness in tertiary HPT have been published. However, for sHPT, it was demonstrated that uncontrolled HPT increases the economic burden due to higher medication and hospitalization costs [36], and PTx is more cost-effective than treatment with cinacalcet after 15 months of treatment [37]. Considering the increasing survival of kidney transplant patients, one could assume PTx to be even more cost-effective in patients with tHPT, especially when considering that in dialysis patients, the cinacalcet costs for one QALY exceed $ 100,000 [38].

Some limitations of this study should be addressed. First, the retrospective design makes this study susceptible to bias as demonstrated by the difference at baseline between our groups. There is no clear guideline on indications for PTx, so indications may differ between participating centers. The PTx group was younger and had higher PTH levels at baseline. But though the baseline PTH levels were higher in the PTx group, the effect was more profound compared with that in the cinacalcet group.

Second, in the Netherlands, KT patients are followed in the transplant center in the first year after transplantation. If uneventful, follow-up will then be performed at the referring hospital, with only annual follow-up at the transplant center. Thus, some outcome data after the first post-transplant year was incomplete. Only the short-term outcomes of the first post-transplant year have therefore been reported.

Third, outcomes in this study are primarily of biochemical nature. Comparison of clinical outcome parameters (e.g., cardiovascular disease or bone mineral density values and fracture risks) was not possible due to lack of BMD measurements and lack of follow-up at our centers.

A minority of patients in our cohort received vitamin D supplementation, recommended in the KDIGO guideline CKD-MBD as a first-line treatment for HPT since 2003 [6]. However, our cohort encompasses over 20 years of treatment, including the years up to 2003. This is not applicable for the group of patients treated with cinacalcet, as cinacalcet was registered after the first KDIGO guideline. This undertreatment with vitamin D might have resulted in overtreatment with cinacalcet in the cinacalcet group. Vitamin D levels would have been important to consider, as vitamin D stimulates intestinal calcium reabsorption which, in turn, inhibits the release of PTH from the parathyroid through negative feedback to the calcium-sensing receptor. As a consequence, higher vitamin D levels would lead to higher calcium and suppressed PTH levels and vice versa. Unfortunately, vitamin D levels were not available for our study. However, the majority of patients (66.0%) used vitamin D supplements at baseline, and there was no significant difference in vitamin D supplement use among both groups. Moreover, the strong PTH decline in the PTX group seems independent of vitamin D supplement use, which remained more or less stable during the first year post-PTx (Table 4). Fourth, the higher percentage of patients that reached normocalcemia in the PTx group (100%), compared with the cinacalcet-treated group (67%), in combination with the stronger effect on PTH levels in the PTX group is unlikely to be explained by differences in vitamin D levels between both groups. Lastly, in the present study, side effects of cinacalcet were not reported. These outcomes are often not reported in patient charts, although reporting has been reliable in the EVOLVE trial. In this RCT, 46% of the subjects reported side effects and 18.1% of patients discontinued the treatment [25] In other studies with cinacalcet, the rate of side effects was unfortunately not reported [39, 40].

In conclusion, treatment with cinacalcet in patients with tHPT normalized serum calcium concentrations, but did not lead to normalization of PTH concentration. After parathyroidectomy, both calcium and PTH normalized in the PTx group. Due to the heterogeneity of the two study groups and the retrospective design of this study, definitive recommendations for daily practice cannot be made. Given the effects of high PTH concentrations on renal allograft survival, we favor PTx in all patients with tHPT. Future prospective randomized studies with long-term follow-up are needed to define the role of surgery in patients with tHPT considering clinical outcomes and economic benefits.
